# Genetic Signature of Acute Lymphoblastic Leukemia and Netherton Syndrome Co-incidence—First Report in the Literature

**DOI:** 10.3389/fonc.2019.01477

**Published:** 2020-01-17

**Authors:** Szymon Skoczen, Konrad Stepien, Wojciech Mlynarski, Piotr Centkowski, Kinga Kwiecinska, Michal Korostynski, Marcin Piechota, Elzbieta Wyrobek, Angelina Moryl-Bujakowska, Wojciech Strojny, Magdalena Rej, Jerzy Kowalczyk, Walentyna Balwierz

**Affiliations:** ^1^Department of Oncology and Hematology, University Children's Hospital, Krakow, Poland; ^2^Department of Pediatric Oncology and Hematology, Institute of Pediatrics, Jagiellonian University Medical College, Krakow, Poland; ^3^Student Scientific Group of Pediatric Oncology and Hematology, Jagiellonian University Medical College, Krakow, Poland; ^4^Department of Pediatrics, Oncology, Hematology and Diabetology, Medical University of Lodz, Lodz, Poland; ^5^Department of Molecular Neuropharmacology, Institute of Pharmacology of Polish Academy of Sciences, Krakow, Poland; ^6^Intelliseq sp. z o.o., Krakow, Poland; ^7^Department of Pediatric Hematology, Oncology and Transplantology, Medical University of Lublin, Lublin, Poland

**Keywords:** Netherton syndrome, malignancy, leukemia, children, mutation

## Abstract

The aim of the following case report is to provide a description of acute lymphoblastic leukemia (ALL) in a patient with Netherton syndrome (NS). A 15-year-old male with NS was referred with suspicion of acute leukemia. Severe anemia, leukocytosis, thrombocytopenia, and elevated CRP level were demonstrated in pre-hospital laboratory tests. Physical examination revealed generalized ichthyosiform erythroderma. ALL was diagnosed on the basis of bone marrow biopsy. The patient was initially classified as CNS3 status. No signals indicating fusion of *BCR/ABL1, ETV6*, and *RUNX1* genes and *MLL* gene rearrangement were found in the cytogenetic analysis. The patient was qualified for chemotherapy and treated according to ALL IC-BFM 2009 protocol for high-risk ALL. During induction therapy, severe skin toxicity occurred (WHO grade III), which prompted the modification of treatment down to intermediate-risk strategy. In the course of reinduction therapy, severe chemotherapy-induced adverse drug reactions occurred, including progression of skin toxicity to WHO grade IV. The patient achieved complete remission. In view of life-threatening toxicities and the confirmed complete remission, intensive chemotherapy regimen was discontinued and maintenance treatment was started. Because of the baseline CNS3 status, the patient received cranial radiotherapy. Whole exome sequencing (WES) was used to identify disease-associated mutations. WES revealed two germline mutations: a novel premature termination variant in *SPINK5* (p.Cys510^*^), along with a novel potentially pathogenic variant in *NUP214* (p.Arg815Gln). Somatic mutations were known pathogenic variants of *JAK2* (p.Arg683Gly), *IL17RC* (p.Ala303Thr), and potentially pathogenic non-synonymous variants of *TTN* (p.Gly1091Arg and p.Pro17245Leu), *ACTN2* (p.Ile143Leu), *TRPV3* (p.Arg729^*^), and *COL7A1* (p.Glu2842fs) genes. Currently, the patient continues maintenance chemotherapy, with stable status of skin lesions and no features of ALL relapse. To our knowledge, this is the first report of ALL in a patient with NS. As has been presented, in such patients, optimal treatment according to the current protocols is extremely difficult. WES was used to confirm the diagnosis of Ph-like ALL in our patient. The detection of *JAK2* gene mutation offers the possibility of therapy personalization. A specific signature of rare germline variants and somatic mutations can be proposed as a factor predisposing to the co-incidence of ALL and NS.

## Background

Netherton syndrome (NS) (OMIM #256500) is a rare autosomal recessive disorder. It is caused by mutations of Kazal type 5 (*SPINK5*) gene, encoding a serine protease inhibitor LEKTI (1, 2). Its incidence is estimated at 1:200,000 births (3) and ~200 cases described in the literature. It is one of ichtyosis syndromes, a clinically and genetically heterogeneous group of disorders of cornification (4). Typical examples include the triad of congenital ichthyosiform erythroderma, trichorrexis invaginata (a hair shaft abnormality), and lesions resembling atopic dermatitis, but phenotypic features are variable in individual patients. The impairment of the epidermal barrier leads to thermoregulatory dysfunction, fluid and electrolyte disturbances, as well as frequent recurrent skin and respiratory infections. Other features may include intellectual disability, neurological deficits, and growth retardation.

The issue of carcinogenesis in NS is still a matter of debate. According to current knowledge, NS does not belong to syndromes with proven genome instability (5). However, in most cases, a higher incidence of skin and mucous membrane tumors was described, particularly in early adolescence (6–9). Several hypotheses were proposed to explain this phenomenon. In one patient, human papillomavirus (HPV) DNA was preferentially detected in malignant lesions, and it was speculated that impaired epidermal defense mechanisms could have promoted latent HPV DNA persistence in the skin (10). Recurrent infections typical for NS may suggest that immunodeficiency, including abnormalities of memory B cells and natural killer cells, which are common in NS, might be associated with cutaneous carcinogenesis (11). Moreover, long-term ultraviolet (UV) and immunosuppressive therapies used in the treatment of patients with NS may promote the development of cancer in adulthood (7). Importantly, no direct relationship between LEKTI deficiency and skin cancers has been proven so far.

The association of NS and hematological malignancies is a different and very rare issue. To date, only a few cases of coexistence of acute leukemia, the most common childhood malignancy, with various types of inherited ichthyoses have been described (12). According to our knowledge, no case report of acute leukemia in patients with NS has been published to date and possible mechanisms of such a coincidence are not known. The suspected causes included an effect of immunodeficiency, an increased chromosome fragility and breakage rate, or a simple matter of chance (12). However, in previous case reports, high-throughput genome sequencing methods have not been used to exclude the genetic background of the coexistence of these disorders.

## Case Presentation

We report a 15-year-old male diagnosed with NS in infancy and referred to the Department of Pediatric Oncology and Hematology with suspected acute leukemia. His medical history was remarkable for high-grade fever (up to 40°C) lasting 1 week. A day before admission, syncope occurred immediately after rising from bed. Based on alarming symptoms and patient's congenital disease, a complete blood count and C-reactive protein (CRP) were performed by a primary care physician. Severe anemia (hemoglobin level 3.8 g/dl), leukocytosis (white blood cell count 14,000/μl), thrombocytopenia (platelet count 16,000/μl), and elevated CRP level (63 mg/dl) were found. Physical examination revealed hyposthenic body habitus, growth retardation, and generalized ichthyosiform erythroderma.

Bone marrow biopsy was performed as a part of routine surveillance, and it revealed 98% of lymphoblasts. Common type acute lymphoblastic leukemia (ALL) was diagnosed and was qualified as L1 subtype according to FAB classification. Immunophenotype examination of bone marrow cells revealed the presence of population of abnormal lymphoblasts (about 98%) presenting SSClow and low expression of CD45 (CD45-dim). A normal male karyotype (46,XY) was found in the bone marrow cytogenetic analysis. Split-signal-FISH of bone marrow cells showed no *BCR/ABL1* fusion genes. No *MLL* gene rearrangements as well as *ETV6* and *RUNX1* fusion genes were found. No additional validation of FISH negative results was performed. Due to the high level of suspicion of central nervous system involvement and intraretinal hemorrhages, the patient was classified as CNS3 status at baseline. Cerebrospinal fluid examination revealed no lymphoblasts. In addition, a high IgE level of 10,700 IU/ml was found.

**Figure 1:**
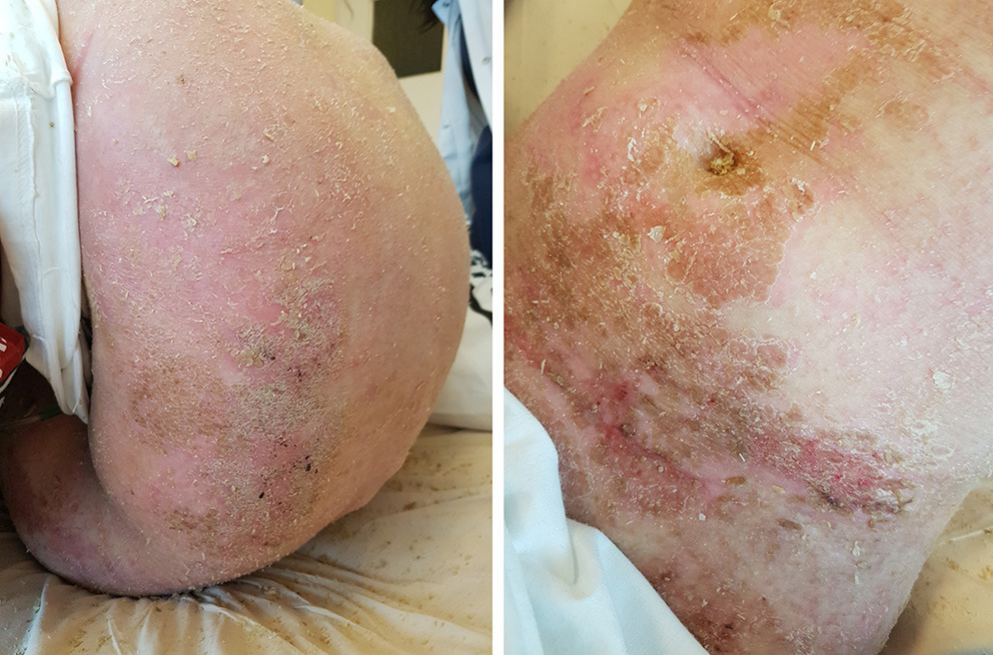
Generalized ichthyosis linearis circumflexa on the patient's trunk.

**Figure 2:**
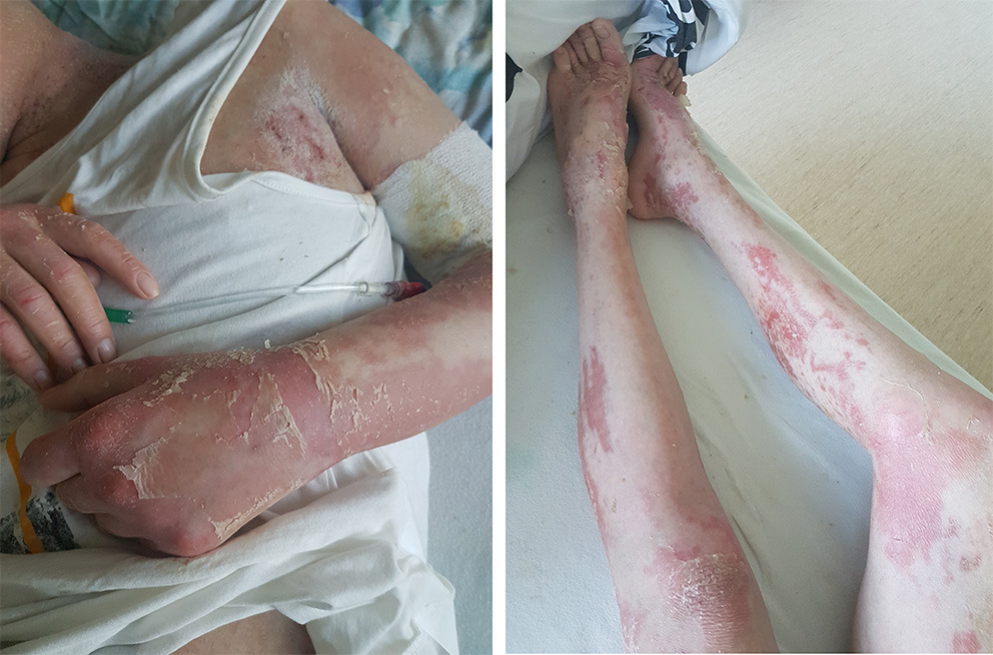
Large erythematous plaques and intensive scaling on the patient's limbs.

The treatment according to ALL IC-BFM 2009 protocol was introduced. A satisfactory response to glucocorticoid prophase was seen. Bone marrow aspiration on day 15 revealed 1.5% blasts and minimal residual disease (MRD) of 11%. Complete remission with MRD of 0.087% was achieved on day 33. According to the treatment protocol, the assessment of MRD on day 15 is crucial for qualification of a patient to a specific risk group. Based on this result, the patient was stratified as high-risk group and an appropriate chemotherapy regimen was started. During the induction phase, severe skin toxicities appeared (WHO grade III), which prompted the modification of treatment down to intermediate-risk strategy. The patient received induction, early intensification, consolidation (3 of 4 methotrexate cycles), and an initial phase of reinduction (until day 19). In the course of chemotherapy, severe adverse drug reactions occurred: skin toxicity (WHO grade IV: Figures 1, 2), glucocorticoid-induced diabetes, hepatotoxicity, syndrome of inappropriate antidiuretic hormone hypersecretion (SIADH), as well as recurrent infections. After initial reinduction, the complete remission was confirmed with negative MRD result. Due to the life-threatening toxicities and in view of achieving a complete remission, intensive chemotherapy was discontinued and maintenance treatment was introduced. Considering the initial CNS3 status and the risk of central nervous system infection caused by repeated lumbar punctures, therapeutic cranial radiotherapy in the dose of 18 Gy in 12 fractions was used. Moreover, the negative MRD status was additionally confirmed.

Currently, 2 years from the start of ALL treatment, the patient's general health status is good. Maintenance chemotherapy is continued with stable skin lesions and no signs or symptoms of ALL relapse.

### Infectious Complications

At initial evaluation, positive IgG antibodies against *Toxoplasma gondii* and Epstein-Barr virus (EBV) viral capsid antigen (VCA) were detected.

In view of immunodeficiency associated with NS, the patient received prophylactic phenoxymethylpenicillin, co-trimoxazole, and antifungal agents throughout the treatment period. Nevertheless, conjunctivitis and sinusitis occurred during the treatment, with *Pseudomonas aeruginosa* etiology confirmed in both cases, and nasopharyngeal colonization with this pathogen was also found.

The patient was febrile several times. At the beginning of consolidation, he was admitted with high-grade fever approaching 39.5°C due to progression of dermatitis. An extended course of low-dose oral prednisone as a treatment for dermatitis was continued until the third cycle of methotrexate. Topical treatment included emollients, hydrocortisone-tanin, and hydrocortisone-cholesterol-based ointments. Repeated problems with central venous access occurred due to NS-related dermatitis. A Hickman central venous catheter was implanted into the right internal jugular vein but healing was incomplete and removal of the catheter was necessary. After the third methotrexate cycle, the patient presented with signs and symptoms of sepsis with fever up to 40°C. *Staphylococcus haemolyticus* was cultured in blood samples taken from the catheter. The catheter was removed and another one was implanted, followed by subsequent implantation of several peripherally inserted central catheters (PICC). These also required removal as *P. aeruginosa, S. haemolyticus, Staphylococcus epidermidis*, and *Enterococcus faecium* were subsequently detected in blood cultures. Swab cultures revealed saprophytic bacterial flora with *Staphylococcus simulans, S. epidermidis*, and *Enterococcus faecalis*. Because of sepsis with fever up to 39°C, fungal pneumonia, and diarrhea, the patient received only an initial phase of reinduction chemotherapy.

### Description of Laboratory Investigations and Genetic Tests

Genetic tests were performed to explore the possible genetic basis of NS and ALL coexistence. Genomic DNA was extracted from bone marrow cells collected at ALL diagnosis according to the standard protocols. WES was performed on Illumina HiSeq X Ten platform, using sequencing libraries generated with Agilent SureSelect All Exon V6 Kit and TruSeq DNA Library Preparation Kit. The obtained 16.6 giga-bases of aligned sequence data resulted in >200× mean coverage of the target capture regions. The raw reads from FASTQ files were aligned to hs38DH reference genome using BWA (version 0.7.15). Resulting BAM files were post-processed with Sentieon software implementing the equivalent GATK 3.5 Best Practices protocol, and the GVCFs files were generated. The personalized panel of genes was created based on Human Phenotype Ontology (HPO) (version: April 13, 2017). The panel consists of genes associated with the phenotype: “HP:0006721 Acute lymphoblastic leukemia;” “HP:0008064 Ichthyosis,” and “ORPHA:634, OMIM:256500 Netherton syndrome” (385 genes total). SNVs (single nucleotide variants) and INDELs (short insertions and deletions) were discovered via joint genotyping of GVCFs.

The resulting VCF was annotated and filtered using an analysis of designed workflow. After discarding variants with QUAL < 30, remaining variants were annotated on variant and gene level. The variant level annotations consisted of flags designating the presence of variant OMIM, PubMed, or HGMD databases), names of diseases associated with a given variant and their clinical significance [ClinVar (version: April 4th, 2017) and MITOMAP (version: January 19th, 2017) databases], and their frequencies in 1000 Genomes, ExAC, gnomAD, MITOMAP, and local database. Variants were also annotated with impact prediction scores: SnpEff annotations (version: 4.3i), SIFT—deleteriousness score (version: February 2th, 2017), and GERP and PhastCons—evolutionary conservation scores. Variant-level filtering removed variants more frequent than 1% in 1000 Genomes, ExAC, or MITOMAP populations and variants with SnpEff impact LOW or MODIFIER. Gene-level filtering retained variants located in genes present in the panel.

Among the genes associated in HPO database with autosomal dominant model of NS, ALL, and ichthyosis inheritance, there were 10 significant variants identified. Most of those mutations have high SIFT deleteriousness score (<0.11) and high evolutionary conservation (GERP > 4.7 and PhastCons = 1). A novel premature termination germline variant in *SPINK5* (p.Cys510^*^) was identified. Three variants have been detected in ALL-related genes: a known pathogenic somatic variant in *JAK2* gene (p.Arg683Gly) coding a non-receptor tyrosine kinase, a known pathogenic somatic variant of *IL17RC* (p.Ala303Thr), and a previously non-described germline variant in *NUP214* (p.Arg815Gln). In addition, two different missense variants in *TTN* gene associated with titin isoform N2-B (p.Gly1091Arg and p.Pro17245Leu) as well as a novel potentially significant variant in *ACTN2* (p.Ile143Leu) were identified. There were also two interesting variants in *TRPV3* gene (p.Arg729^*^) in a highly conserved region (GERP > 4.0 and PhastCons = 1) as well as in *COL7A1* (p.Glu2842fs) with lower level of conservation. WES also detected a remaining variant in *RPL15* gene (p.Arg189Gln) associated with Diamond-Blackfan anemia. Results of the analysis are presented in abbreviated form in Table 1.

**Table 1:** Selected somatic and germline genetic variants detected in the patient.

**Gene symbol**	**Position of sequence change**	**Reference SNP ID number**	**Variant type**	**Classification according to ACMG**
*SPINK5*	p.Cys510^*^	–	Germline	Pathogenic
*JAK2*	p.Arg683Gly	rs1057519721	Somatic	Likely pathogenic
*NUP214*	p.Arg815Gln	rs749833713	Germline	Uncertain significance
*IL17RC*	p.Ala303Thr	rs145516404	Somatic	Pathogenic
*TTN*	p.Gly1091Arg	rs72647870	Somatic	Benign
*TTN*	p.Pro17245Leu	rs754702040	Somatic	Likely benign
*ACTN2*	p.Ile143Leu	–	Somatic	Uncertain significance
*TRPV3*	p.Arg729^*^	rs11654533	Somatic	Uncertain significance
*COL7A1*	p.Glu2842fs	rs566181351	Somatic	Uncertain significance
*RPL15*	p.Arg189Gln	rs764132899	Somatic	Uncertain significance

## Discussion of the Underlying Pathophysiology and Significance of the Case

Our case report is related to the diagnosis and treatment of ALL in a patient with NS, which, to our knowledge, has not been reported to date. As presented above, several modifications of the treatment regimen due to exacerbations of skin lesions were necessary. Moreover, numerous infectious complications were seen, which also impeded the oncological treatment. WES analysis revealed the presence of several significant genetic mutations that may explain the etiology of ALL in patients with NS.

Despite the initial stratification of the patient to the high-risk group, it was necessary to apply an intermediate-risk ALL treatment protocol and subsequently to prematurely terminate the intensive phase of the treatment. As has been demonstrated, rigorous adherence to the treatment regimens designed for appropriate risk groups is associated with excellent outcomes (13). Conversely, non-compliance with chemotherapy protocols causes an increased risk of relapse (14–16). Long-term follow-up will be necessary in our patient due to this undertreatment and unknown outcomes of ALL in NS.

Severe skin toxicity was one of the most important factors limiting optimal ALL treatment in the presented case. We hypothesized that the intensive chemotherapy protocol predisposed our patient to extensive skin lesions development. Currently, NS treatment is exclusively symptomatic and is based on the use of emollients, keratolytic agents, antibiotics, and histamine H1 receptor antagonists (17). Corticosteroids of moderate strength as well as calcineurin inhibitors should be used on limited body areas, because of the high risk of systemic absorption (18, 19). Recent reports concerning the successful use of intravenous immunoglobulins (11) and infliximab (20) appear to represent new therapeutic options. The extensive use of these agents along with the emerging new treatment options will probably reduce chemotherapy-induced skin toxicity in NS who need oncological treatment and allow the use of more aggressive treatment strategies.

Recurrent infectious complications were another problem that occurred during the ALL treatment. A number of different locations were affected and various alarming pathogens were involved. The issue of recurrent infections in NS has been repeatedly reported in the literature. Their frequency has been estimated at ~30% (21). In recent years, Renner et al. (11) have shown reduced memory B cells and decreased NK cell cytotoxicity. Moreover, treatment with intravenous immunoglobulins resulted in clinical improvement and temporarily increased NK cell cytotoxicity. Hannula-Jouppi et al. (22) in a study conducted in 11 Finnish patients with NS confirmed the previous observations on NK cell immaturity and dysfunction as well as reduced levels of memory B cells. Additionally, elevated serum IgG4 levels were detected, which has not been reported previously. Interestingly, both studies presented data concerning impaired post-vaccination immune response in NS (11, 22). The above results have led to the inclusion of NS in the current classification of primary immunodeficiencies (23).

According to the literature, up to 10% of cancer cases that occur in children are associated with well-characterized predisposing genetic syndromes (24). However, the low incidence of genetic factors in the etiology of childhood leukemia and absence of NS in leukemia-related syndromes suggest the pure coincidence of both diseases (25). The results of WES should be interpreted with extreme caution. We have recently demonstrated its effectiveness in the assessment of a pediatric patient with coincidence of two cancers (26). The detected variant in the *SPINK5* gene is a genetic confirmation of the inherited NS. Several other mutations have also been considered as cancer-related. Therefore, we decided to discuss their potential impact on our patient's disease.

A novel variant in *SPINK5* gene as a germline mutation was detected in our patient. The discovered p.Cys510^*^ mutation is associated with occurrence of premature termination codon. Based on a recent large systematic review, about 80 different mutations in 144 families have been described so far. Interestingly, genotypes with mutations located more upstream in LEKTI correlate with more severe phenotypes, which may explain the wide variety of clinical phenotypes of NS (1). Most of the mutations described so far have also been associated with premature termination (4). The use of genome sequencing technology provides a unique opportunity to identify novel potentially pathogenic variants.

*TRPV3* is another skin-specific gene whose mutation was detected in our patient. Activation of *TRPV3* in skin keratinocytes causes skin barrier formation, hair growth, wound healing, temperature sensing, itch, and pain perception (27). Its dysfunction leads to the occurrence of Olmsted syndrome (28), allergic dermatitis (29), and also promotes the formation of scars (30). Potentially, the coexistence of *SPINK5* and *TRPV3* mutations could lead to a more aggressive course of NS, but so far, this relationship has not been described in the literature.

WES analysis also detected several leukemia-related genes. It should be emphasized that the number of comprehensively characterized whole genomes of ALL patients is relatively small. Janus kinase 2 (JAK2) is a member of the non-receptor tyrosine kinase family. *JAK2* activating mutations occur in 8.6% *BCR/ABL1*-negative, high-risk pediatric ALL cases (31). Majority of the detected somatic mutations, including our patient, involved the 683rd amino acid residue, which is an important amino acid for the JH2 domain-mediated negative auto-regulation of JAK2 activity (32). The second somatic mutation associated with the JAK pathway regulation was a variant of interleukin-17 receptor (*IL17RC*) gene (33). Nucleoporin 214 (NUP214) is required for cell cycle and nucleocytoplasmic transport. Its abnormalities are found rarely in pediatric leukemias, mostly in T-ALL patients. Five fusion genes associated with *NUP214* have been described so far (34). Their occurrence is associated with worse prognosis due to the chemotherapy resistance (35). In our patient, the germline mutation in *NUP214* (p.Arg815Gln) was found. Based on the Clinvar database, ribosomal protein L15 mutation (*RPL15*) is found in Diamond-Blackfan anemia. This syndrome is associated with hematological malignancies such as myelodysplastic syndrome and acute myeloid leukemia (36). In our patient, however, phenotypic manifestations of the detected mutation were not found (37).

Considering the negative *BCR/ABL1* fusion result in the cytogenetic test and the presence of pathogenic *JAK2, IL17RC, NUP214*, and *RPL15* mutations in the WES, the diagnosed leukemia can be initially qualified as the recently identified Philadelphia chromosome-like acute lymphoblastic leukemia (Ph-like ALL). Sequencing studies have shown that Ph-like ALL represents a complex genomic landscape with diverse genetic alterations. Unfortunately, we did not perform the analysis of prognostically significant *IKZF1* alterations in the current case report (38). Ph-like ALL prevalence is ~12% in children and 21% in adolescents (39). Most patients with Ph-like ALL have positive MRD after remission induction (40). In a large analysis of 1,725 childhood, adolescent, and young adult ALL cases, Ph-like ALL was associated with event-free and overall survival rates equal or inferior to high-risk ALL subtypes (41). The improvement of the unfavorable prognosis can be achieved by the introduction of targeted therapy. Preclinical studies with patient-derived xenograft cells show the high efficacy of JAK2 inhibitors. Ruxolitinib and momelotinib suppress cells with *JAK2* mutations. Moreover, momelotinib also inhibits cells without these mutations, which may suggest some additional mechanisms of action (42). It may also be effective in patients with *IL17RC* mutations. Preliminary clinical observations indicate high effectiveness of these agents. Roberts et al. (41), using genetic methods, identified a small subgroup of high-risk patients with Ph-like ALL in whom targeted therapy was subsequently used. Of the 12 patients who began therapy (including 3 patients treated with ruxolitinib), rapid and sustained responses were found in 11 patients for whom follow-up data were available. According to expert opinions, JAK2 inhibitors may be considered as an add-on, but not as substitutes for chemotherapy in patients with *JAK2* mutations (42). Possibly, an inclusion of a JAK2 inhibitor in a standard maintenance chemotherapy regimen might also improve the prognosis in our patient. However, prospective, randomized, multicenter trials are necessary for approval of the novel targeted drugs in pediatric population.

## Concluding remarks

To our knowledge, we present the first case report of ALL in a patient with NS. As has been shown, optimal treatment according to current protocols is extremely challenging in such patients. WES allowed for detection of two novel germline mutations and initial qualification of leukemia diagnosed in our patient as Ph-like ALL. Moreover, detection of *JAK2* gene mutation may offer an opportunity to personalize the treatment. A specific signature of rare germline variants and somatic mutations can be proposed as a predisposing factor to the coexistence of ALL and NS.

## Data Availability Statement

The datasets for this article are not publicly available in order to maintain anonymity. Requests to access the datasets should be directed to Konrad Stepien, konste@interia.eu.

## Ethics Statement

Ethical review and approval was not required for the study on human participants in accordance with the local legislation and institutional requirements. Written informed consent to participate in this study was provided by the participants' legal guardian/next of kin. Written informed consent was obtained from the minor(s)' legal guardian/next of kin for the publication of any potentially identifiable images or data included in this article.

## Author Contributions

SS, KS, PC, and WB contributed to case report concept and design. SS, KS, and PC wrote sections of the manuscript. WM, KK, MK, MP, EW, AM-B, WS, and MR performed diagnostic tests and collected relevant clinical data. SS, WM, MK, AM-B, WS, JK, and WB critically revised the article. All authors were responsible for the integrity and accuracy of the data and approved the submitted version.

### Conflict of Interest

The authors declare that the research was conducted in the absence of any commercial or financial relationships that could be construed as a potential conflict of interest.
